# Recent advances in graphene-based biosensor technology with applications in life sciences

**DOI:** 10.1186/s12951-018-0400-z

**Published:** 2018-09-22

**Authors:** Janire Peña-Bahamonde, Hang N. Nguyen, Sofia K. Fanourakis, Debora F. Rodrigues

**Affiliations:** Department of Civil and Environmental Engineering, University of Houston, Houston, TX 77204-4003 USA

**Keywords:** Nano-biosensors, Graphene, Graphene oxide, DNA, Antibody, Enzyme, Detection, Pathogens

## Abstract

Graphene’s unique physical structure, as well as its chemical and electrical properties, make it ideal for use in sensor technologies. In the past years, novel sensing platforms have been proposed with pristine and modified graphene with nanoparticles and polymers. Several of these platforms were used to immobilize biomolecules, such as antibodies, DNA, and enzymes to create highly sensitive and selective biosensors. Strategies to attach these biomolecules onto the surface of graphene have been employed based on its chemical composition. These methods include covalent bonding, such as the coupling of the biomolecules via the 1-ethyl-3-(3-dimethylaminopropyl) carbodiimide hydrochloride and *N*-hydroxysuccinimide reactions, and physisorption. In the literature, several detection methods are employed; however, the most common is electrochemical. The main reason for researchers to use this detection approach is because this method is simple, rapid and presents good sensitivity. These biosensors can be particularly useful in life sciences and medicine since in clinical practice, biosensors with high sensitivity and specificity can significantly enhance patient care, early diagnosis of diseases and pathogen detection. In this review, we will present the research conducted with antibodies, DNA molecules and, enzymes to develop biosensors that use graphene and its derivatives as scaffolds to produce effective biosensors able to detect and identify a variety of diseases, pathogens, and biomolecules linked to diseases.

## Background

Conventional sensing methods, such as lateral flow immunoassay, fluorescent microarray and electrochemical methods, polymerase chain reaction (PCR)-based methods, DNA microarrays, DNA sequencing technology, enzyme-linked immunosorbent assay (ELISA), among others [1–6] require expensive reagents, high-precision instruments, and quantification methods to achieve highly sensitive detection. Additionally, most of the reactions cannot be monitored quantitatively in real-time. Thus, novel sensors that are simple, inexpensive, and possess highly specific sensing properties would allow for the assessment of target biomolecules in real-time, which would have broad clinical applications.

Sensors in medicine and life sciences have been used to monitor vitals, diagnose patients, and improve the critical care of patients [7–10]. Due to the need for early detection and diagnosis of diseases, as well as minimally invasive detection approaches, many novel sensors have been developed. A particular focus of sensor development has been in miniaturization via application of nanomaterials to fabricate nanosensors. The nano-sized nature of nanomaterials and their unique chemical and electrical properties can improve patient care by making the sensors minimally invasive and extremely sensitive [10]. While the sensitivity of the sensors is critical in detecting their target molecule, the accuracy and detection limit of the sensors are also critical parameters as they can influence their positive and negative predictive values. Typically, studies report the linear range of biosensors, which can give the detection limit of the sensor. However, due to the novelty of recent sensor designs there are no detailed reports or statistics related to accuracy, precision, positive, and negative predictive values of these parameters. Future studies should take into consideration these important parameters.

**Fig. 1:**
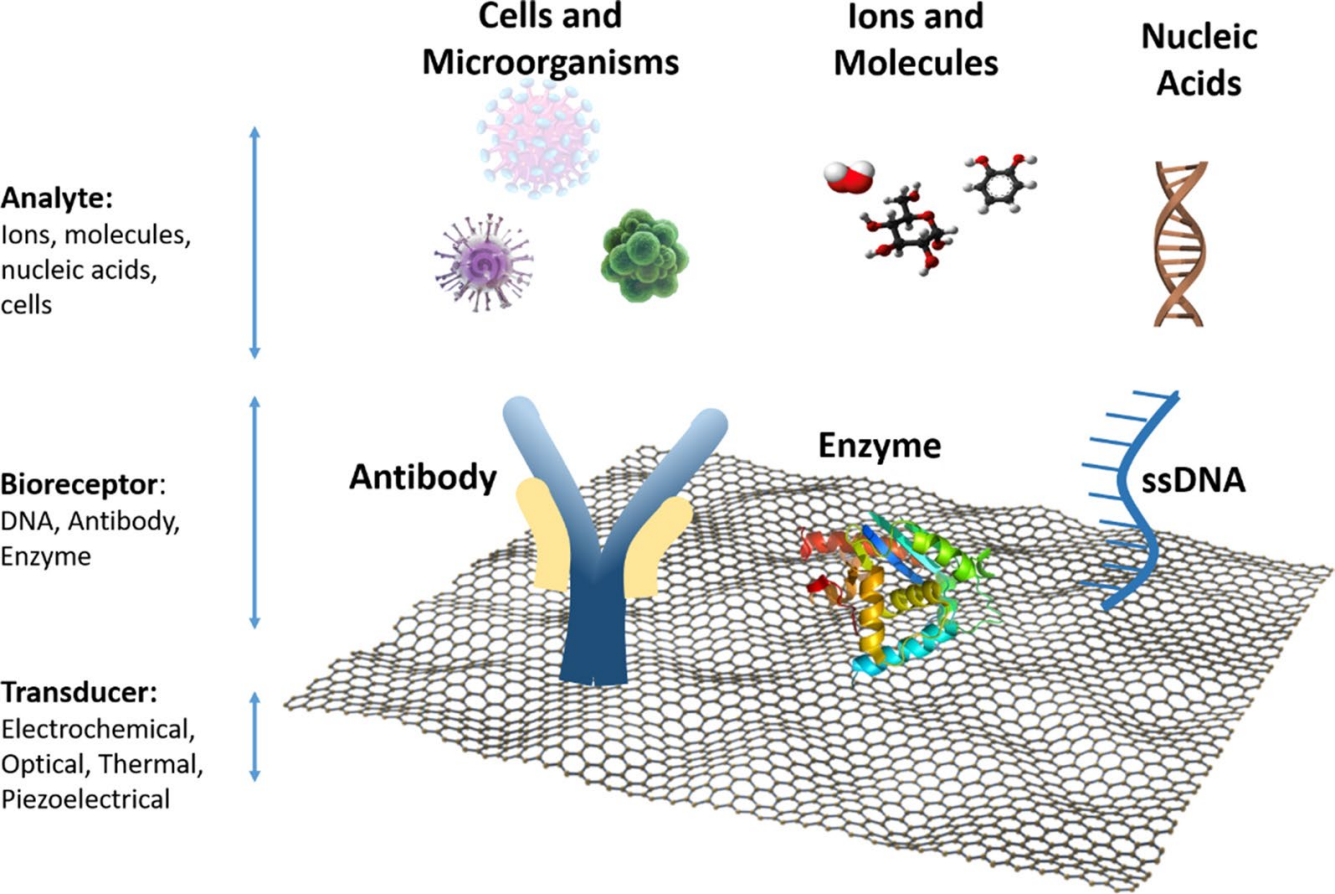
Examples of biosensors and components on a graphene platform

Among the nanomaterials used for nano-sensor fabrication, graphene and graphene-based nanomaterials have been showing the most promise since they present an enhanced signal response in a variety of sensing applications [11–13]. Furthermore, graphene-based nanomaterials possess high surface area and offer excellent biocompatibility with a variety of biomolecules, like antibodies, enzymes, DNA, cells, and proteins [13]. The incorporation of such biological molecules in graphene’s detection scheme (Fig. 1) has allowed the development of the so-called biosensors. These biosensors can detect multiple molecules, biomolecules and even cells [14, 15].

## Graphene-based nanomaterials as a biosensor

**Fig. 2:**
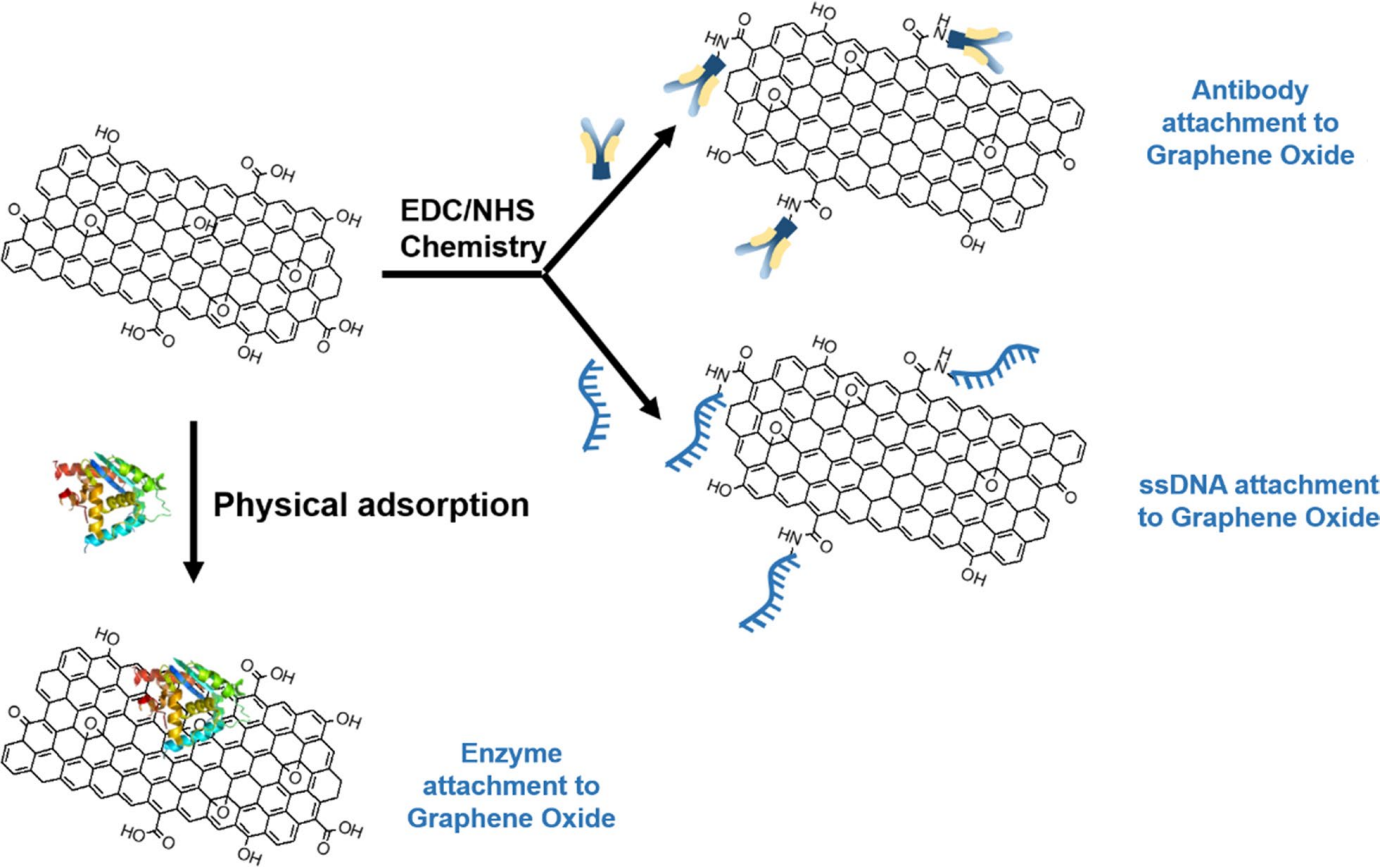
Schematic of the most common attachment methods of bioreceptors, such as antibody, DNA and enzymes onto graphene surfaces

In general terms, sensors consist of two elements: a receptor and a transducer (see Fig. 1). The receptor is the organic or inorganic material that interact specifically with the target molecule. The target molecule can be organic, inorganic or even whole cells. The transducer is the part of the sensor, which converts chemical information into a measurable signal. Graphene-based nanomaterials are used as transducers of biosensors, which are involved in converting the interactions between the receptor and the target molecules into detectable measurements [16]. For this to occur, the bioreceptor (molecules such as antibodies, ssDNA, and enzymes) needs to be attached to the transducer surface. The most common attachment method used for antibodies and ssDNA immobilization onto graphene and its derivatives (graphene oxide, reduced graphene oxide) is EDC/NHS chemistry, while enzymes are most commonly immobilized using physisorption (see Fig. 2).

Graphene has been employed in the design of different biosensors of various transduction modes because of its large surface area, electrical conductivity, high electron transfer rate and capacity to immobilize different molecules [17]. For instance, the conjugated structure of graphene can facilitate the electron transfer between the bioreceptor and transducer, which can generate high signal sensitivity for electrochemical sensors [12, 16, 18, 19]. Furthermore, graphene-based nanomaterial can act as a quencher in the transducer to generate fluorescent biosensors. Studies have determined that graphene (G), graphene oxide (GO), and reduced graphene oxide (rGO) have a very high efficiency of fluorescent quenching [20–22].

When using graphene nanomaterials for designing sensors, some aspects of the graphene properties affecting the detection limit of the target molecules need to be taken into consideration. For instance, different synthesis batches of graphene and derivatives, as well as different synthetic methods can lead to different properties and functionalities of the graphene-based nanomaterials in the biosensors. The orientation between the G, GO or rGO sheets and the bioreceptor can also directly affect the selectivity and sensitivity of the biosensors. Additionally, the number of layers, the functional groups and oxidation states of graphene and derivatives will cause differences in the sensing performance among the sensors and even impact the bonding between the transducer and bioreceptor. The amount of functional groups on the nanomaterials can also affect the interactions and the detection limit of the target molecule. In this context, it is often necessary to block any nonspecific adsorption sites on the nanomaterial to prevent unspecific binding of biomolecules instead of the target molecules. This can be accomplished by coating with blocking reagents such as bovine serum albumin (BSA) [23], casein, or superblock [24], or treating the sensor with tween surfactant [25]. By taking into consideration these limitations, biosensors of graphene-based nanomaterials can have high sensitivity/stability as well as fast response time, potentially resulting in advances in healthcare and diagnosis.

In this mini-review, we will briefly summarize recent developments on biosensor technology with graphene and graphene-based nanomaterials. More specifically, we will focus on antibody, DNA and enzyme-based biosensors with applications in life sciences as well as in clinical settings. We aim to present conceptual advances that have been made in the synthesis and applications of biosensors for clinical diagnosis and real-time molecular detection.

## Graphene-based nanomaterials and antibodies

**Fig. 3:**
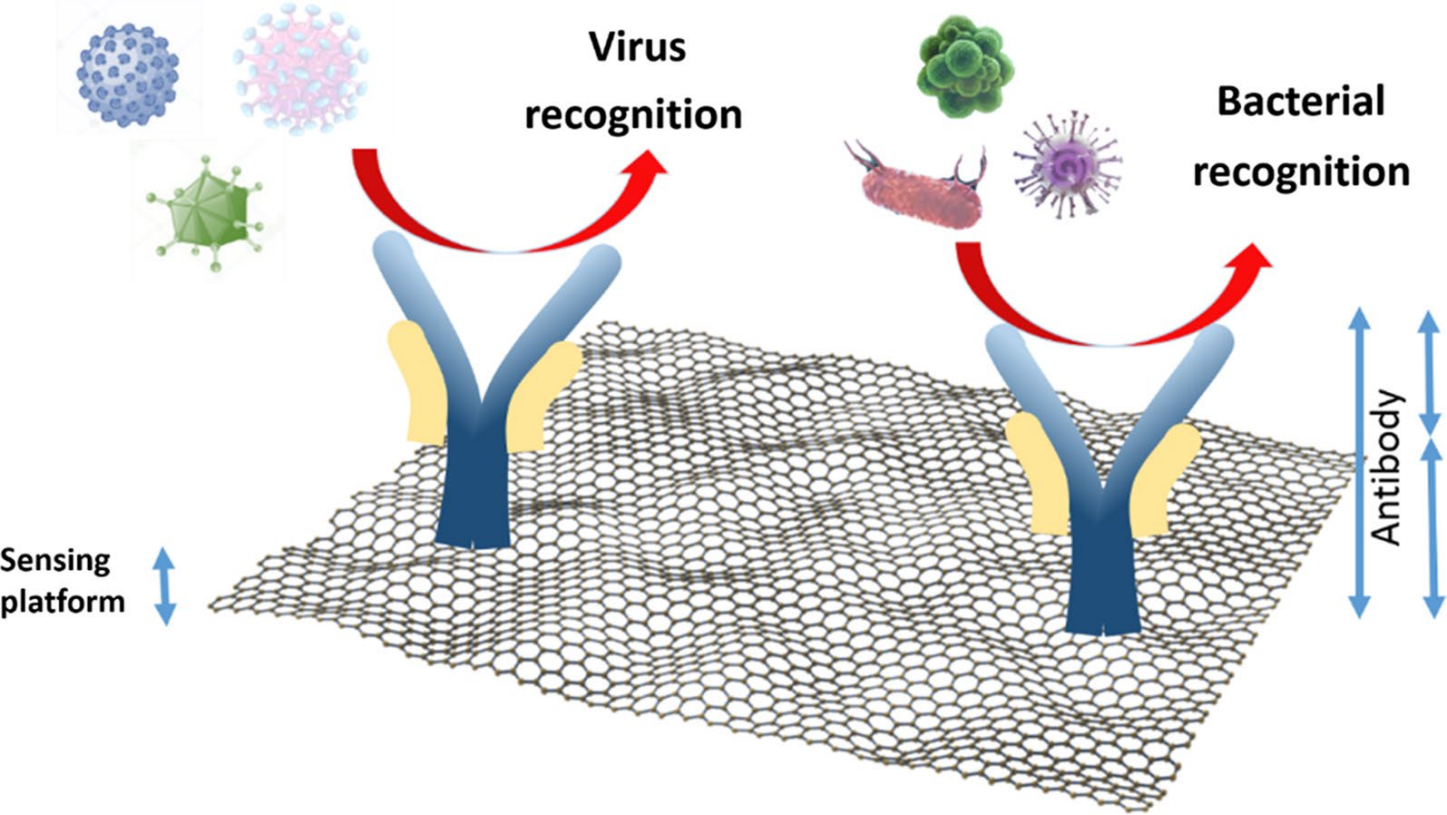
Scheme of graphene modified with antibodies for the recognition of pathogens

The analytical detection platforms that measure the specific conjugation reaction between antibody and antigen are called immunosensors. The biocompatibility and high-affinity binding of antibodies to antigens make this molecule attractive for use in several fields, particularly in diagnostics. The antibody (Ab) structure is made of four polypeptide chains with a characteristic “Y” shape (Fig. 3). The chains are connected via a single disulfide bond. The structure of the Ab consists of two different parts: the “arms” of the Ab that contain two domains, i.e. a constant and a variable domain. The variable domain gives the selectivity of antibodies to a specific antigen. The “body” of the Ab part consists of two different segments, the crystallizable fragment (Fc) and the antigen-binding fragment (Fab). The Fc and Fab contain carboxyl (–COOH) and amino (–NH_2_) groups that bind to the target molecule with high affinity [26, 27]. This high-affinity recognition to a specific antibody–antigen reaction is mainly because of the structure, properties, and reactivity of the antibodies, making them excellent candidates for sensing applications.

The versatility of functional groups of the GO surface allows different strategies for Ab attachment. The Ab functionalization can be summarized in Table 1. Most of the strategies to functionalize GO with antibodies involve functionalization via 1-ethyl-3-(3-dimethylaminopropyl) carbodiimide hydrochloride (EDC)/*N*-hydroxysuccinimide (NHS) (EDC/NHS) chemistry reaction, electrostatic bonding, or via 1-pyrenebutanoic acid succinimidyl ester (PASE) linker. The functionalization via EDC/NHS chemistry is the most popular and versatile method for producing biochemical conjugations. EDC is a water-soluble cross-linker agent, which allows direct bioconjugation between carboxyl and amine groups. In this reaction, the nucleophilic attack from the primary amine group from the antibody forms an amide bond with the carboxyl groups on the GO surface. This process can form conjugates between two different molecules with an amide group [28].

**Table 1:** Overview of discussed graphene antibody-based nanosensors

Target	Immunosensor design	Detection methods	Antibody	Antibody binding	Detection limit	Refs.
*Escherichia coli*	Graphene oxide cellulose nanopaper	Photoluminescence	Antihuman IgG Ab	Conjugation process	1.60 ng/mL	[50]
	Graphene/PMMA	Electrical	Anti *E. coli* O157:H7 antibody	–	10 CFU/mL	[38]
	Graphene	Electrical	Anti-*E. coli* antibody	Via PASE linker	10 CFU/mL	[31]
	Graphene	Electrical	Anti-*E. coli* O157:H7 antibodies	Via PASE linker	10–10^7^ cells/mL	[32]
	Reduced graphene oxide	Electrical	Generic anti-*E. coli* antibody	EDC–NHS chemistry	10^3^ CFU/mL	[37]
*Salmonella typhimurium*	GO–AgNPs nano composite	Cyclic voltammetry	Anti-*S. typhimurium*	EDC–NHS chemistry	10 CFU/mL	[39]
Zika virus	Graphene	Electrical	Anti-Zika NS1	NHS surface chemistry	0.45 nM	[33]
Dengue virus	Graphene oxide	Electrochemical impedance spectroscopy	4G2 monoclonal antibody	Electrostatic bond	0.12 PFU /mL	[34]
Adenovirus	Graphene quantum dots	Optoelectronic	Anti-adenovirus, Group II (HEV) polyclonal antibody	Electrostatic bond	8.75 PFU/mL	[51]
Avian influenza virus H7	Gold nanoparticle–graphene nano composites (AuNPs–G)	Electrochemical immunosensor	H7-polyclonal antibodies and H7-monoclonal antibodies	EDC/NHS chemistry	1.6 pg/mL	[41]
Influenza A virus	Graphene oxide-MB–chitosan	Electrochemical	Monoclonal antibodies (H5N1 or H1N1)	Covalent and crosslinked via chitosan	9.4 pM and 8.3 pM	[48]
Cholera toxin	Graphene–polypyrrole	Surface plasmon resonance	Anti-CT	π–π interactions	4 pg/mL	[52]
Rotavirus	Graphene oxide	Photoluminescence	Rotavirus antibodies	Carbodiimide-assisted amidation reaction	10^5^ PFUmL	[35]
Hepatitis C virus	Graphene quantum dots with silver nanoparticles	Electrochemical immunosensing	Anti-HCV antibody	NH_2_ group of antibody was covalent attachment to the AgNPs	3 fg/mL	[40]
HIV	Peptide- functionalized UCNPs to graphene oxide	Fluorescence	Anti-HIV-1 gp120 antibody	π–π interactions	2 nM	[47]
Celiac disease	Polyamidoamine dendrimer with GQDs on AuNP embedded in MWCNT	Electrochemical	Anti-tTG antibody	EDC/NHS chemistry	0.1 fg per 6 µL	[46]
Alzheimer disease	Magnetic core-plasmonic shell nanoparticle attached hybrid graphene oxide	Surface-enhanced Raman spectroscopy	Cy3 antibody	Amine functionalization	100 fg/mL	[44]
Cardiovascular diseases	Graphene oxide	Electrochemical	PAC1 antibody	EDC/NHS chemistry	–	[36]
Hormones	Reduced graphene oxide	Electrochemical	Anti-GHRL and anti-PYY	EDC–NHS chemistry	1.0 pg/mL GHRL and 0.02 pg/mL PYY	[53]
Cancer	Magnetic Fe_3_O_4_@GO composites	Electrochemical	RAB0331 for PSA and Lifeome Biolabs/Cusabio EL008782HU-96 for PSMA	EDC–NHSS	15 fg/mL for PSA and 4.8 fg/mL for PSMA	[45]
	Graphene–PYR–NHS	Electrochemical impedance spectroscopy	Monoclonal antibody anti-carcinoembryonic antigen	Non-covalent modification	less than 100 pg/mL	[54]
	Reduced graphene and gold nano particle	Electrochemical	Anti-estradiol antibody (curve)	EDC–NHS	0.1 fmol	[42]
	Reduced graphene oxide gold nano particle	Electrochemical	p53 antibodies	Electrostatic interactions	0.088 pg/mL	[43]
	β-cyclodextrin functionalized graphene nanosheet	Electrochemical	CEA primary antibody (Ab1), and CEA secondary antibody (Ab2)	EDC–NHS	20 fg/mL	[49]

The detection of the target molecules can be achieved through different methods (see Table 1). The most commonly described method is electrochemical. Electrochemistry is a method that measures any electrical or chemical changes at the electrode/electrolyte interface. This method is based on the conformational changes produced by the biorecognition between the antibody and the antigen. These nanosensors consist of a working electrode (where the reaction takes place) and a reference electrode (which makes the connection to the electrolyte and allows the current to flow between the two electrodes). Electrochemical sensors include the measurement of current, potential or resistance where the electrode transducer is able to detect the change in the electrical signal caused by the binding reaction [29]. This method is selected over other immunosensor methods since it is simple, rapid, sensitive, uses small sample volumes, and presents good selectivity [26]. This method, however, has a few limitations, such as binding affinity and irreversible antigen–antibody interaction [30].

Graphene-based nanomaterials on antibody biosensors offer a broad versatility regarding pathogen detection. Recently, several graphene-antibody biosensors with clinical applications have been developed for early detection of diseases (Table 1). Antibody nanosensors with G were developed to detect *E. coli* [31, 32] and Zika virus [33]. GO, on the other hand, has been employed for the detection of dengue virus [34], rotavirus [35] and cardiovascular diseases [36]. rGO has been employed to detect *E. coli* in different samples [37] but with higher detection limits comparing to G [31, 32] and G modified with poly(methyl methacrylate) (PMMA) [38]. More advanced research has shown that the modification of G with nanoparticles can improve the sensing properties of the transductor. In this context, G has been modified with silver nanoparticles for the detection of *Salmonella typhimurium* [39] and hepatitis C virus (HCV) [40]. Gold nanoparticles attached to G surfaces have been employed to detect avian influenza virus H7, [41] and for diagnosis, prognosis, and prediction of treatment efficacy and recurrence of cancer [42, 43]. The modification of G with magnetic nanoparticles allows the early detection of Alzheimer [44] and also cancer diagnosis [45]. More complex biosensors modifying the surface of G with dendrimer [46], polymers [47, 48] or cyclodextrin [49] have been developed to detect Celiac disease, HIV, Cholera toxin, and cancer. Table 1 shows in more detail the design of these immunosensors, their detection method, detection limit, as well as the antibody used to detect their particular target molecule. Immunosensor have been developed for different types of microbes, such as bacteria and viruses, as well as diseases. In bacterial detection, graphene and graphene oxide as sensor platforms give the lowest detection limit (10 times less), compared to reduced graphene oxide. For virus, the modification of graphene with gold and silver nanoparticles by covalent attachment of the antibody allows the detection of concentrations as low as picograms per mL (pg/mL) of virus. In the case of detection of cancer cells, the modification of graphene oxide by functionalization with magnetic Fe_3_O_4_ allows to detection limits in femtograms (fg). An overall comparison among all currently available sensing platforms indicates that the functionalization of graphene or graphene oxide with silver, gold or other metal nanoparticles and the antibody attachment via covalent bond, typically allows the lowest detection limits.

The early detection of these diseases with such sensors can aid in diagnosis, prevention, and management of the disease in ‘high-risk’ individuals, which in turn would contribute to better management and survival of patients. Many biosensors based on graphene nanomaterials have been proposed in the last few years for the diagnosis and real-time monitoring of the health status of patients. While the limitations of these types of sensors (binding affinity and irreversible antigen–antibody binding) are not fully rectified, the proposed biosensors exhibit very low detection limits (see Table 1), speed, sensitivity, and selectivity making these graphene-based biosensors ideal candidates for medical diagnostic tests.

## Graphene-based nanomaterials and deoxyribonucleic acid (DNA)

Deoxyribonucleic acid (DNA) has a broad range of physical, chemical, and biological properties making this biomolecule highly suitable for biosensor technologies. Among the most critical properties of DNA for a biosensor is its flexibility, easy synthesis, facile chemistry to attach to diverse platforms, simple regeneration and high specificity due to unique sequences of nucleotides [55, 56]. However, several advantages and disadvantages of DNA biosensors have been identified. Significant advantages of DNA biosensors include high specificity, ability to be used for real time analysis, to be designed as a small measurement system, and to perform multiplex measurements of different targets [57, 58]. However, one of the major disadvantages of DNA biosensors is that DNA can be easily degraded, thus, requiring specific storage and analysis conditions, such as particular media or a buffer to keep the DNA stable and maintain its attachment to the transducer. Additionally, DNA-based sensors’ effectiveness can be affected by changes in pH or temperature [59]. For instance, the sensitivity of DNA biosensors depends on experimental temperatures because the hybridization event of the probe with the target molecules will occur at optimum temperatures to be determined prior to the deployment of the sensor. In the case of pH, the current response shows the highest signal at pH 7.0, while there is almost no signal at pH below 7.0. Therefore, a buffer with potassium or sodium phosphate is needed to enhance the effectiveness of the sensor [60, 61]. Despite their disadvantages, nucleic acids have gained increasingly more attention in the fields of biosensors and biological assays for their applications in genetics, infectious diseases, and detection of pathogens in clinical settings [62]. In DNA biosensors using graphene-based nanomaterials as transducers, there are two main types of sensors: electrochemical and fluorescent sensors.

**Fig. 4:**
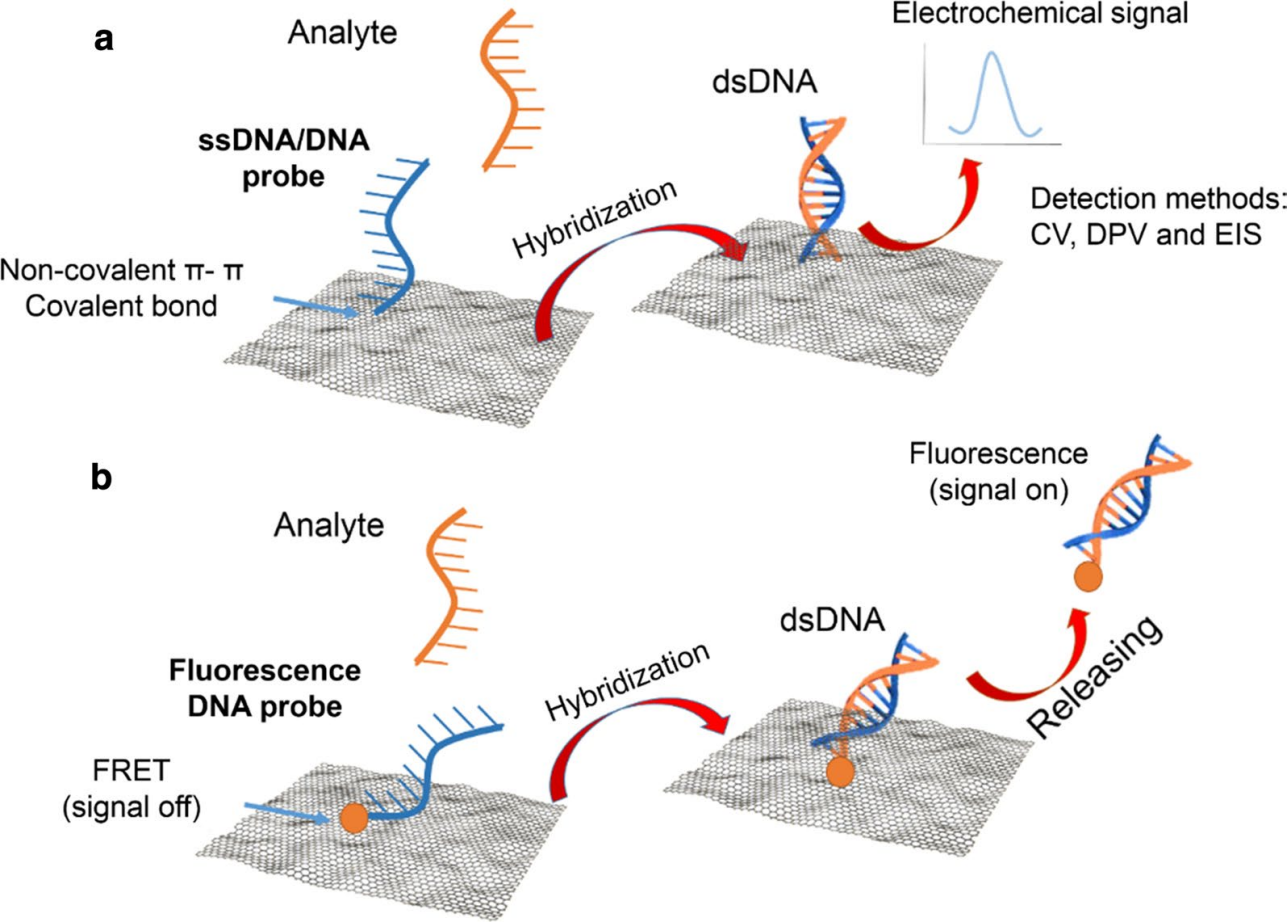
Scheme of graphene-based nanomaterials as a DNA biosensor. Electrochemical detection (**a**) and fluorescent detection (**b**)

The electrochemical sensor is based on measurements of the change in voltage, current, or impedance that can result from changes in electrochemical factors, such as electron loss, conductivity or capacitance changes, which are caused by the hybridization of DNA or the oxidation of adenine (A), thymine (T), cytosine (C) and guanine (G) of the DNA. The electrochemical signals produced by these biosensors can be detected using cyclic voltammetry (CV), differential pulse voltammetry (DPV) or electrochemical impedance spectroscopy (EIS) [18, 63]. In the electrochemistry approach, the immobilization of DNA is done via π–π interactions on the surface of graphene-based nanomaterials (Fig. 4). G edges and GO or rGO with their functional groups (carboxylic, hydroxyl and epoxide groups) can also be used to covalently interact with the DNA [19, 64]. The most common chemistry used for immobilization of the DNA on graphene-based nanomaterials is EDC/NHS, which is described in detail in the antibody section. Research to improve sensitivity and selectivity of electrochemical biosensors have been mostly in the modification of the transducers. For instance, the original glassy carbon electrode (GCE) can be modified with GO for the direct detection of A, T, G, and C for dsDNA or ssDNA using the DPV method at pH 7.0 [65]. In another study, the GCE was modified with rGO and DNA probes to hybridize with a target DNA to be detected with either EIS or CV [25]. This study takes advantage of the large surface area and high conductivity of rGO. Another study investigated the DNA sensor using the sharp and active edges of reduced graphene nanowalls (RGNW) to detect dsDNA with a sensitivity ranging from 0.1 fM to 10 mM. In this study, the authors suggest that the active edge sites of the RGNW sheet could enhance the electron transfer between DNA and the electrode in the DPV more uniformly [66]. Depending on the sensing material and target, the sensor can have a wider detection range and sensitivity. For example, in the case of dsDNA detection, the best material identified in the literature is graphene nanowall, which can sense quantities as low as 0.1 fM (Table 2). For ssDNA, the modification of reduced graphene oxide sensors with labeled ssDNA and gold nanoparticles (ssDNA–AuNPs–ERGO) increases the sensitivity to a lower detection limit of 0.005 fM (Table 2) [67]. Graphene-based DNA biosensors have been investigated with focus on lowering the detection limits, speeding time of measurements and facilitating the fabrication process and biomedical applications. Therefore, there has been a large number of published studies to improve these features of graphene-based DNA biosensors, which are summarized in Table 2.

**Table 2:** Graphene-based DNA biosensors with electrochemical detection

Detected element	Sensing material	Detection range	Refs.
dsDNA ssDNA	Graphene nanosheets Graphene nanowalls	2.0 pM to less than 10 mM 0.1 fM to 10 mM	[66]
dsDNA	Epitaxial graphene	1 µM	[68]
BRCA1 DNA	Graphene/Au	1 fM	[69]
*Staphylococcus aureus nuc* gene sequence	CTS–Co_3_O_4_–GR/CILE (Chitosan–Co_3_O_4_–graphene–carbon ionic liquid electrode)	1.0 × 10^−12^ to 1.0 × 10^−6^ M with the detection limit as 4.3 × 10^−13^ M	[70]
dsDNA	Thionine–graphene nanocomposite (Thi–G)	1.0 × 10^−12^ to 1.0 × 10^−7^ M and low detection limit at 1.26 × 10^−13^ M	[71]
Survivin gene	Graphene–nanostructure gold nanocomposite film glassy carbon electrode (G-3D Au/GCE)	50–5000 fM detection limit at 3.4 fM	[72]
dsDNA	[Co(phen)2(Cl)(H_2_O)]^+^ AuNPs/GR (gold–graphene) modified electrode	2.50 × 10^−11^ to 1.25 × 10^−9^ M Detection limit at 8.33 × 10^−12^ M	[73]
ssDNA	Graphene analogue tungsten sulfide–graphene (WS2–Gr) composite	0.0–500 pM Detection limit at 0.0023 pM	[74]
Multidrug resistance (MDR) DNA	Nitrogen-doped graphene nanosheets functionalized with Au nanoparticles (N–G/Au)	Detection limit 3.12 × 10^−15^ M	[75]
ssDNA	Nitrogen-doped graphene (NG) and Fe_3_O_4_ nanoparticles	1.0 × 10^−14^ to 1.0 × 10^−6^ M Detection limit 3.63 × 10^−15^ M	[76]
ssDNA of HIV-1 gene	Graphene–Nafion composite film	Detection limit 2.3 × 10^−14^ M	[77]
DNA	AuNCs/GR nanobybrids and exonuclease III (Exo III) aided cascade target	0.02 fM to 20 pM Detection limit at 0.057 fM	[78]^23^
ssDNA	Graphene and polyaniline nanowires (PANIws) modified glassy carbon electrode	2.12 × 10^−6^ to 2.12 × 10^−12^ M Detection 3.25 × 10^−13^ M	[79]
dsDNA, ssDNA and single nucleotide polymorphism	Poly(amidoamine) dendrimer (PAMAM) with graphene core	1 × 10^−6^ to 1 × 10^−12^ M Detection limit 1 pM	[80]
ssDNA	Electroactive dye azophloxine functionalized graphene nanosheets (AP–GNs)	1.0 × 10^−15^ to 1.0 × 10^−11^ M Detection limit at 4.0 × 10^−16^ M	[81]
ssDNA	Gold nanorods decorated GO sheets (Au NRs–GO)	1.0 × 10^−9^ to 1.0 × 10^−14^ M Detection limit at 3.5 × 10^−15^ M	[82]
Hepatitis B virus (HBV)	GO/pencil graphite electrode (GO/PGE)	20 to 160 µg/mL Detection limit 2.02 µM	[83]
DNA	GO–Chitosan (CHI) nano-composite	10 fM to 50 nM Detection limit 10 fM (60 s hybridization times) and 100 fM at 25 °C	[84]
ssDNA	ssDNA-Fe@AuNPs-AETGO	1.0 × 10^−14^ to 1.0 × 10^−8^ M Detection limit 2.0 × 10^−15^ M	[85]
ssDNA	rGO-graphene double-layer electrode	10^−7^ to 10^−12^ M Detection limit 1.58 × 10^−13^ M	[86]
MDR1 gene	Au nanoparticles/toluidine blue–graphene oxide (Au NPs/TB–GO)	1.0 × 10^−11^ to 1.0 × 10^−9^ M Detection limit 2.95 × 10^−12^ M	[87]
DNA	AuNPs/ERGNO/GCE	2.0 × 10^−7^ to 1.0 × 10^−6^ M Detection limit at 1.0 × 10^−6^ M	[88]
ssDNA	ssDNA–AuNPs–ERGO	1 × 10^−17^ M to 1 × 10^−13^ M Detection limit 5 aM	[67]
ssDNA	Gold nanoparticles decorated rGO (Au NPs/rGO)	0.1 µM to 0.1 fM Detection limit at 35 aM	[89]
*Listeria monocytogenes*	Au/GR/CILE	1.0 × 10^−12^ to 1.0 × 10^−6^ M Detection limit 2.9 × 10^−13^ M	[90]
Amelogenin gene (AMEL)	rGO modified glassy carbon electrode (GCE/RGO)	1.0 × 10^−20^ to 1.0 × 10^−14^ M Detection limit 3.2 × 10^−21^ M	[25]
Methicillin-resistant *Staphylococcus aureus* (MRSA) DNA	rGO-modified glassy carbon electrode	10^−13^ M	[91]
ssDNA	Thionine functionalized rGO (Thi–rGO)	1.0 × 10^−17^ to 1.0 × 10^−12^ M Detection limit 4.28 × 10^−19^ M	[92]

The fluorescent DNA nanosensor is based on the hybridization of two single-stranded DNA (ssDNA). One ssDNA is labeled with a fluorescent dye, and the other is the complementary DNA corresponding to the target DNA. This method requires optical detection; therefore it takes advantage of the optical quenching property of graphene-based materials to enhance the visualization and detection of the target ssDNA [12]. The immobilization of the fluorescent-labeled DNA can be carried out by direct adsorption of the DNA probe on the graphene-based surface through the π–π interaction between the ring structure of the DNA bases and the graphene surface.

One example of fluorescence biosensors that has been developed is the GO-based sensor. This sensor has been produced with multicolor DNA probes for detecting different sequence-specific DNA. This multiplex GO-based DNA sensor presents low background fluorescence and excellent emission signal from specific targets when the hybridization occurs [93]. Another widely use of the fluorescence sensing approach, which can also employ graphene-based materials, is the fluorescence resonance energy transfer (FRET or Förster). In this detection method, initially, the fluorescent labeled DNA probe is quenched to the graphene-based nanomaterials surface through FRET, making the fluorescent signal off (Fig. 4). Upon hybridization of the probe with the target DNA, the fluorescent molecule is released with the dsDNA from the graphene surface, and the fluorescent signal is turned on for optical detection [16]. For instance, in the effort to propose a reliable, biocompatible and scalable biosensor for HIV-1 detection, a nanocomposite of gold nanoparticles (AuNPs) and GO was synthesized and used as a quencher with the use of fluorescent carbon dots (CDs) and a DNA probe, also called nano quencher. The FRET strategy was also used in the CDs/AuNPs/GO nanoprobe. In the presence of target ssDNA, hybridization occurs, and the fluorescent signal turns on. The presence of AuNPs on the GO nanosheets serves to quench the fluorescence of CDs in the absence of the target DNA. AuNPs/GO exhibits exceptional selective and sensitive capability in the DNA biosensors [94]. This sensor has a detection limit as low as 15 fM. In the effort to find the best sensor, different composites of graphene-based materials have been used to achieve the desired sensitivity. For instance, ssDNA can be detected with a fluorescent graphene sensor with a sensitivity as low as 0.5 pM using target recycling Exonuclease III (Table 3). Table 3 presents the summary of other studies taking advantage of the quenching ability of graphene-based nanomaterials to enhance or improve the fluorescent detection of DNA biosensors.

**Table 3:** Graphene-based DNA biosensors with fluorescent detection

Detected element	Sensing material	Detection range	Refs.
ssDNA	GO	Detection limit 200 nM	[95]
ssDNA	GO and exonuclease III	Detection limit 20 pM	[96]
ssDNA	GO	200 nM	[97]
DNA and exonuclease activity	GO ethidium bromide (EB)	50 to 2500 nM Detection limit 32 nM	[98]
*Staphylococcus aureus* DNA	GO–DNA sensor	0.0125 to 3.125 nM Detection limit at 0.00625 nM	[99]
Hepatitis B virus (HBV) sequences	GO/pencil graphite electrode (GO/PGE)	20 to 160 µg/mL Detection limit 2.02 µM	[83]
ssDNA	Exonuclease III (ExoIII) and GO	Detection limit 0.5 pM	[100]
HIV-1 gene	AuNPs/GO nanocomposite	50.0 fM to 1.0 nM Detection limit at 15 fM	[101]
ssDNA	GO	0 to 25 nM Detection limit at 100 pM	[93]
T antigen gene of SV40 DNA	GO	40.0 to 260 nM Detection limit at 14.3 nM	[93]

In summary, the two methods seem efficient and present low detection limits. However, each technique has its advantages and disadvantages, which depends mainly on the ability of immobilization of the DNA in the graphene-based nanomaterials and the method of measurement. The electrochemical detection method takes into account the large surface area and conductivity of the nanomaterials. The detection is based on the types and numbers of bases present in the DNA, which would cause the changes in electrical potential for the measurement. Therefore, homogenous deposition of the probe on the graphene material is essential for accurate measurements. Also, the electrostatic potential and DNA length could affect the efficiency of the sensor. On the other hand, fluorescence detection can be performed in ssDNA or dsDNA regardless of the length of the DNA. This method is based on the quenching and optical ability of graphene-based nanomaterials. One of the main disadvantages of this method is that it can overestimate the fluorescence signal due to the high background fluorescence signal in some complex samples, such as serum samples. On the other hand, the fluorescent-labeled probe can lose its intensity (photobleach) over time. Results of graphene-based DNA biosensor studies have shown that there is still need for further investigations related to the mechanisms of interactions between the DNA probe or modified DNA probe and the graphene-based transducer to provide more reliable and accurate measurements. Such studies could overcome the current disadvantages of the method by lowering the detection limit of the current sensors.

## Graphene-based nanomaterials and enzymes

Enzymes deserve particular attention in biosensor design because they can be easily manipulated and have high stability. Furthermore, these molecules are involved in the metabolism of all organisms; they are reusable and highly selective catalysts that can discriminate between L and R enantiomers in different molecules. Enzymes can catalyze a large number of reactions with high specificity, efficiency, and selectivity, which are essential parameters in sensor design [102]. However, use of enzymes in sensors require modification or careful consideration of the type of enzyme that should be used. Enzyme stability can be problematic as higher temperatures can cause their denaturation resulting in the loss of catalytic activity and reduced sensor functionality. While initially this issue was addressed via the use of thermophilic enzymes, nowadays, thermophilic enzymes are created or modified to become more robust using biological engineering [103]. For instance, to alter enzyme properties, researchers have used site-directed mutagenesis or chemical modifications to improve enzyme stability [103]. Alternatively, with advancements in recombinant DNA technology, enzymes can be manipulated rapidly by cloning and overexpressing the desired enzyme gene [103]. This approach has solved several issues related to enzymatic stability and specificity.

Advancements in enzyme-based biosensor research have resulted in improved stability while reducing enzymatic loss and enzyme response time [104]. It has been demonstrated that the stability of enzymes is affected by pH, ionic strength, chemical inhibitors, solvent polarity, and temperature. The structure of graphene-based nanomaterials can be an effective transducer since it allows the direct electron transfer between enzymes and electrodes [19]. Furthermore, graphene-based materials have been shown to be excellent substrates for increasing thermal stability, enzymatic activity, and for enzyme immobilization [105–107].

Several approaches have been developed to immobilize enzymes onto graphene surfaces to create enzyme-based biosensors. Some of the most common methods are sonication, mixing, ultrasound, and cyclic voltammetry. These methods allow the attachment of the enzymes via adsorption, covalent bonding, or physical entrapment. To date, the nonspecific binding of the enzyme to graphene via physical adsorption is the most common one (see Table 4) since this immobilization technique is chemical-free and straightforward. Another method used to immobilize enzymes on the nanomaterial is the EDC/NHS chemistry. This method described earlier is also common for enzymes because of its high stability and robustness.

**Table 4:** Recent studies using graphene-based materials to immobilize enzymes

Enzyme	Immobilization platform	Testing compound	Detection method	Attachment	Range	Refs.
Laccase, HRP	Fe_3_O_4_–rGO	–	–	Adsorption	–	[109]
Laccase	GO–rhodium nanoparticles	17β-estradiol	Electrochemical	Donor–acceptor interactions	0.9–11 pM	[110]
Laccase	Palladium–copper nanocages on rGO	Phenol	Electrochemical	Adsorption	0.005–1.155 mM, 1.655–5.155 mM	[111]
Laccase	Yolk shell Fe_2_O_3_	2,6-dimethozyphenol	Electrochemical	Gluaraldehide reaction	0.025–750 μM	[112]
Laccase	Graphene–cellulose microfiber	Catechol	Amperometric	Adsorption	0.085–209.7 μM	[113]
Laccase	MoS_2_ and graphene quantum dots	Caffeic acid	Electrochemical	Electrostatic interaction	0.38–100 μM	[126]
HRP	CaCO_3_ microspheres encapsulated with a graphene capsule	Hydrogen peroxide	Electrochemical	Absorption	0.01–12 mM	[114]
HRP	3D graphene/methylene blue-carbon nanotubes	Hydrogen peroxide	Electrochemical	In-situ self-polymerized polydopamine	0.2 μM–1.1 mM	[127]
Bilirubin Oxidase	Electrochemically reduced GO	–	–	Adsorption	–	[116]
GOx	ZnS–graphene	Hydrogen peroxide, glucose	Electrochemical	–	–	[117]
GOx	Silk–graphene field effect transistor	Glucose	Electrical	Hydrophobic interaction	0.1–10 mM	[118]
GOx	Nanostructured graphene with conducting polyaniline	Glucose	Electrochemical	Adsorption	10.0 μM–1.48 mM	[119]
GOx	TiO_2_–GO–OISL	Hydrogen peroxide	Electrochemical	Immobilization	1–120 μM	[120]
GOx	Chitosan/Nafion/Pt nanoparticle/SGGT	Hydrogen peroxide, glucose			3–300 μM, 0.5 μM–1 mM	[121]
GOx	GO modified by amidation	Glucose	–	Carbodiimide coupling	–	[122]
GOx	3D GO and PANI	Glucose	Electrochemical	–	0.07–1.10 mM	[123]
GOx	AuPd–rGO–polyimide	Hydrogen peroxide, glucose	Electrochemical	Adsorption	0.004–1.0 mM, 0.024–4.6 mM	[124]
GOx	3D graphene	Glucose	Electrochemical	–	0.3–6 mM	[125]

**Fig. 5:**
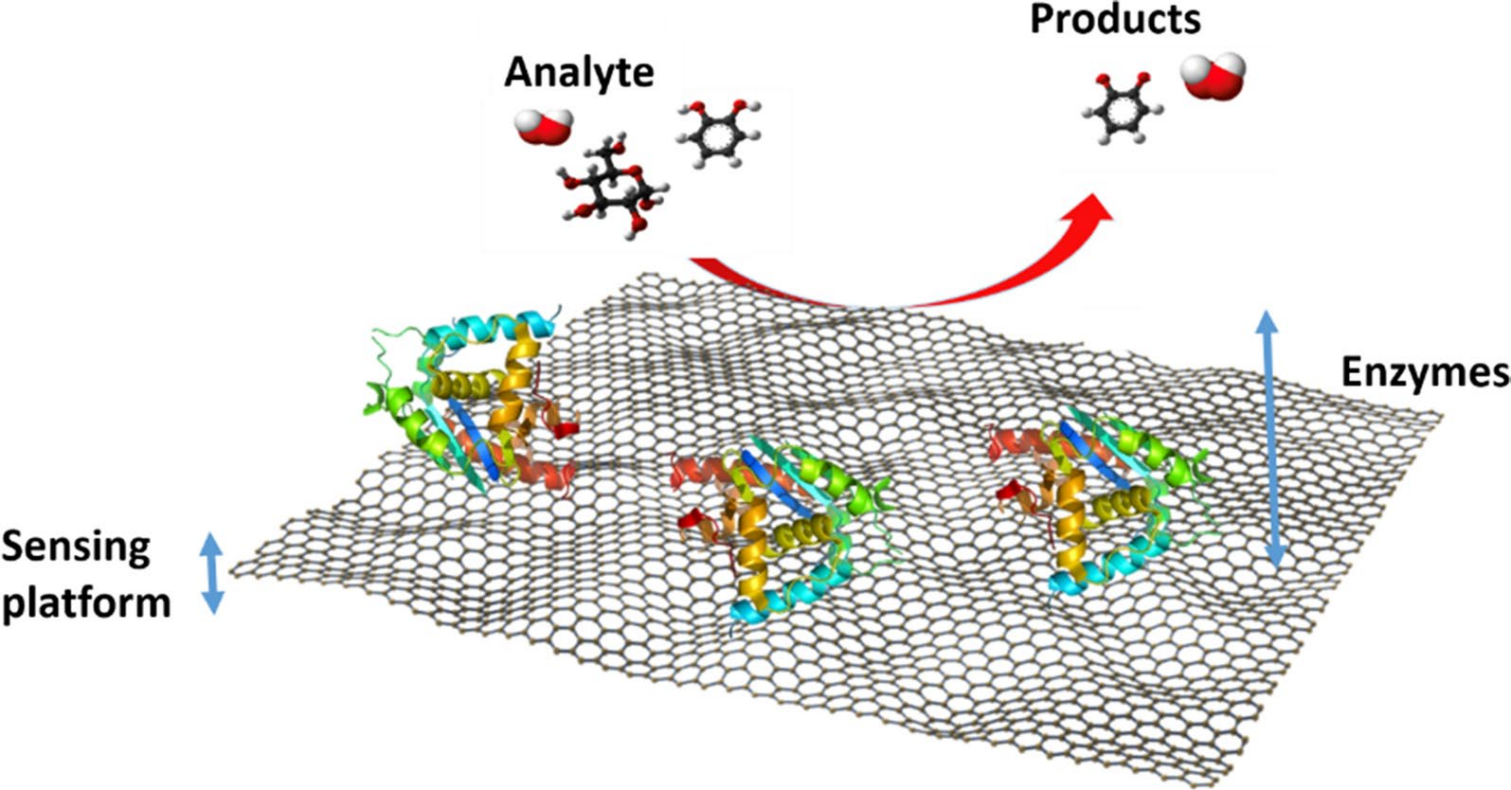
Example of an enzyme biosensor

Enzyme-based biosensors are typically of electrochemical nature. This method possesses advantages over the others because the electrodes can sense materials present in the host without damaging the system. Enzyme-based electrochemical biosensors rely primarily on two mechanisms; one is based on the catalytic properties of the enzymes (the enzyme catalyzes the analyte from its undetectable form to a detectable form), and the other is based on enzyme activity inhibition/moderation [108]. Each of these two mechanisms can create a detectable electrical signal change on the sensor electrode allowing for the quantification of a particular analyte. In particular, this electrical signal is generated from the change in current on the surface of the substrate as a direct result of the enzyme’s activity. Enzymes catalyze redox reactions, which either produce or consume electrons, thus altering the electrical current flowing to the detection platform. The fundamental principle of how enzymatic biosensors work is presented in Fig. 5. While enzymes can be costly to utilize, sensors employing enzymes can detect a variety of compounds with high specificity that would otherwise be difficult to detect in complex mixtures. For example, these sensors can be particularly useful in detecting compounds such as phenols, hydrogen peroxide, 17β-estradiol, glucose, and bilirubin as described later in this section. Table 4 shows a variety of compounds capable of being detected by commonly immobilized enzymes and the resulting detection range achieved by each of the fabricated sensors.

Different molecules have been detected with enzyme-based nanosensors. The most commonly used model enzymes utilized for the development of these sensors are laccase and horseradish peroxidase (HRP) [109]. These enzymes are less costly, more commonly available, and versatile allowing them to be used to detect a high number of different compounds. Laccase is an oxygen-reducing enzyme, which can have a variety of applications. For example, a laccase-based electrochemical biosensor was developed for the detection of 17β-estradiol, a natural hormone classified as an emerging contaminant affecting humans and aquatic life [110]. Additionally, laccase can be used for the detection of phenols and catechols [109, 111–113]. HRP, the other enzyme widely used for enzyme immobilization studies, can help determine hydrogen peroxide concentrations even under complex test conditions [114]. HRP has been immobilized on porous calcium carbonate microspheres encapsulated with graphene capsules and presented high selectivity towards hydrogen peroxide. This sensor platform could potentially be used to immobilize different enzymes for stable, long-term use as a biosensor [114]. Furthermore, HRP, as well as laccase, have been immobilized on a rGO–Fe_3_O_4_ based substrate [109]. This hybrid nanomaterial takes advantage of the properties of rGO and the magnetic properties of iron oxide making it an attractive substrate for biosensor design.

While HRP and laccase have been vital in enzyme biosensor studies, other enzymes can be immobilized to create highly specific biosensors. For example, bilirubin oxidase was immobilized on GO-based surfaces [115, 116]. Such biosensors can have a significant impact in the medical field due to their ability to detect bilirubin, an essential compound for assessing liver function. Another enzyme with medical applications is glucose oxidase (GOx). This enzyme is highly specific and has been used to develop biosensors for the measurement of glucose levels [117–125]. This type of biosensor could be especially important to diabetic patients. As such, in recent years, GOx has been immobilized using different sensing platforms, such as: zinc sulfide decorated graphene [117], three dimensional graphene [125], silk fibroin film on a graphene field effect transistor [118], nanostructured graphene-conducting polyaniline (PANI) composite [119], three-dimensional GO and polyaniline (PANI) composite [123], GO and titanium oxide nanoparticles modified with an organic–inorganic supporting ligand (OISL) [120], and gold–palladium modified polyimide/rGO film [124], among others. Of these platforms the Chitosan/Nafion/Pt nanoparticle/SGGT composite offers the highest sensitivity (down to 0.5 μM) and largest linear range (up to 1 mM) in the detection of glucose [121]. These sensing platforms show the versatility that graphene and its nanocomposites have regarding the chemistry for the detection of different substrates.

## Conclusion

In this mini-review, we have reported recent studies describing graphene and graphene-related biosensors with possible applications in clinical settings and life sciences. We have shown results of the reported analytical performance of each sensor and indicated their use in the life sciences and medical fields. DNA, antibody, and enzyme-based biosensors have been presented in this study since each has its advantages and disadvantages. Overall, the type of sensor selected will depend on the type of application. For example, use of DNA in biosensing technology can be a cost-effective method for the rapid detection of microbes, viruses, or cancer markers. However, due to the vast variety of molecules present in the body, use of antibodies or enzymes in biosensors can be more effective in the detection or monitoring of certain diseases. For instance, antibodies can be used for the specific detection of viruses, such as the Zika virus, HIV, Influenza A virus, among others. Enzymes, on the other hand, have shown to be promising in detecting glucose levels with only small amounts of sample. Overall, the incorporation of graphene and graphene-based nanomaterials in biosensor technologies have shown great promise due to its high surface area, electrical conductivity, electron transfer rate, and its capacity to immobilize a variety of different biomolecules. The development of biosensors that are sensitive, stable, and specific to their target molecule and that can be processed rapidly are promising for use in clinical settings. However, to achieve uniform and reliable results and produce biosensors capable of being used in the medical field, many more studies need to be conducted examining the safety and reliability of the sensors.

Although graphene is an excellent electrode material for sensing applications in the medical field, novel methods for well-controlled synthesis and processing of graphene need more attention and should be investigated in future studies. The current chemical strategies to modify the surface of graphene with biomolecules are effective in targeting specific analytes. Nevertheless, the sensing platform may be further refined to avoid the adsorption of unwanted molecules on graphene and improve the orientation of biomolecules on graphene platforms. Hence, a better understanding of the physics and chemistry at the surface of graphene and the interactions with biomolecules at the interface will play an important role in graphene-based nanosensors.

Additionally, miniaturization and production of compact biosensors for diagnostic purposes is an emergent need in sensor technology since it requires development of reliable, reproducible, and cost-effective sensors with high accuracy, sensitivity, and specificity. Lowering the cost of some of these sensors is necessary to increase usability in remote areas for emergency uses. Furthermore, miniaturization of the sensors can allow rapid detection of virus and bacterial pathogens, as well as use in self-monitoring biological implants to detect serious health conditions. The aforementioned applications in the life sciences will serve to protect lives and improve people’s health. However, considerable work must still be done to ensure, guarantee, and corroborate the biocompatibility and non-toxicity of graphene-based nanomaterials such that their long-term use does not pose any health risk.

## Abbreviations

Ab: antibody
A, T, G, C: adenine, thymine, guanine, and cytosine
AD: Alzheimer disease
AuNPs: gold nanoparticles
BRCA1: breast cancer 1
BSA: bovine serum albumin
CD: celiac disease
CDs: carbon dots
CV: cyclic voltammetry
CEA: carcinoembryonic antigen
CHI: Chitosan
DNA: deoxyribonucleic acid
dsDNA: double stranded DNA
DVP: differential pulse voltammetry
EB: ethidium bromide
EDC/NHS: 1-ethyl-3-(3-dimethylaminopropyl) carbodiimide hydrochloride/*N*-hydroxysuccinimide
EIS: electrochemical impedance spectroscopy
ELISA: enzyme-linked immunosorbent assay
ExoIII: exonuclease III
Fab: the antigen-binding fragment
Fc: crystallizable fragment
FET: field effect transistor
FRET: fluorescence resonance energy transfer
GCE: glassy carbon electrode
GHRL: ghrelin
GO: graphene oxide
GOx: glucose oxidase
GQD: graphene quantum dot
HCV: hepatitis C virus
HIV: human immunodeficiency virus
HRP: horseradish peroxidase
LOD: lower detection limit
MWCNT: multiwall carbon nanotube
NP: nanoparticle
OISL: organic–inorganic supporting ligand
PAMAM: poly(amidoamine)
PANI: polyaniline
PASE: 1-pyrenebutanoic acid succinimidyl ester
PCR: polymerase chain reaction
PMP: platelet-derived microparticle
PMMA: poly(methyl methacrylate)
PYY: peptide YY
RGNW: reduced graphene nanowalls
rGO: reduced graphene oxide
SGGT: solution-gated graphene transistor
SNP: single nucleotide polymorphism
ssDNA: single-stranded DNA
Thi: thionine
